# Improved NGS-based detection of microsatellite instability using tumor-only data

**DOI:** 10.3389/fonc.2022.969238

**Published:** 2022-11-17

**Authors:** Ana Claudia Marques, Carole Ferraro-Peyret, Frederic Michaud, Lin Song, Ewan Smith, Guillaume Fabre, Adrian Willig, Melissa M. L. Wong, Xiaobin Xing, Chloe Chong, Marion Brayer, Tanguy Fenouil, Valérie Hervieu, Brigitte Bancel, Mojgan Devouassoux, Brigitte Balme, David Meyronet, Philippe Menu, Jonathan Lopez, Zhenyu Xu

**Affiliations:** ^1^ SOPHiA GENETICS, Saint-Sulpice, Switzerland; ^2^ Cancer Research Centre of Lyon, INSERM 1052, Centre National de la Recherche Scientifique (CNRS) 5286, University of Lyon, Lyon, France; ^3^ Hospices Civils de Lyon, Biopathology of Tumours, GH Est (GHE) Hospital, Bron, France; ^4^ Hospices Civils de Lyon, Department of Anatomopathology, Lyon-Sud Hospital, Lyon, France; ^5^ Hospices Civils de Lyon, Biochemistry and Molecular Biology Department, Lyon-Sud Hospital, Lyon, France

**Keywords:** microsatellite, next-generating sequencing, tumor-only sequencing, pan-cancer, MSI, Mismatch Repair deficiency, Microsatellite instability

## Abstract

Microsatellite instability (MSI) is a molecular signature of mismatch repair deficiency (dMMR), a predictive marker of immune checkpoint inhibitor therapy response. Despite its recognized pan-cancer value, most methods only support detection of this signature in colorectal cancer. In addition to the tissue-specific differences that impact the sensitivity of MSI detection in other tissues, the performance of most methods is also affected by patient ethnicity, tumor content, and other sample-specific properties. These limitations are particularly important when only tumor samples are available and restrict the performance and adoption of MSI testing. Here we introduce MSIdetect, a novel solution for NGS-based MSI detection. MSIdetect models the impact of indel burden and tumor content on read coverage at a set of homopolymer regions that we found are minimally impacted by sample-specific factors. We validated MSIdetect in 139 Formalin-Fixed Paraffin-Embedded (FFPE) clinical samples from colorectal and endometrial cancer as well as other more challenging tumor types, such as glioma or sebaceous adenoma or carcinoma. Based on analysis of these samples, MSIdetect displays 100% specificity and 96.3% sensitivity. Limit of detection analysis supports that MSIdetect is sensitive even in samples with relatively low tumor content and limited microsatellite instability. Finally, the results obtained using MSIdetect in tumor-only data correlate well (R=0.988) with what is obtained using tumor-normal matched pairs, demonstrating that the solution addresses the challenges posed by MSI detection from tumor-only data. The accuracy of MSI detection by MSIdetect in different cancer types coupled with the flexibility afforded by NGS-based testing will support the adoption of MSI testing in the clinical setting and increase the number of patients identified that are likely to benefit from immune checkpoint inhibitor therapy.

## Introduction

The DNA mismatch repair (MMR) pathway safeguards the genome from base substitution and insertion-deletion (indels) during DNA replication (1). Genetic or epigenetic loss of one or more of the involved proteins results in MMR deficiency (dMMR), leading to increased mutation rates (2).

dMMR is a predictive pan-cancer marker of response to immune checkpoint inhibitor therapy (3, 4) (5). The current standard of dMMR testing is evaluating the expression of the four MMR proteins by immunohistochemistry (IHC) (6). However, IHC tests cannot be combined with other molecular diagnostics, limiting its adoption in cancer types where this molecular phenotype is rare, and false-positive and negative immunostaining results impact their accuracy. Detection of microsatellite instability (MSI), a well-established signature of dMMR (2), is a suitable alternative to IHC (6). Microsatellites (1-6 nucleotide tandem repeat motifs) are informative for dMMR status since their contraction or expansion, resulting from DNA replication errors, are normally repaired by the MMR pathway (7).

In the clinical setting, the most used method to evaluate MSI status analysis of allelic size variation in a panel of five mononucleotide repeats (homopolymers) (6, 8) is using polymerase chain reaction (PCR) followed by capillary electrophoresis. However and despite its widespread use, the analytical performance of this solution in cancers other than colorectal cancer, for which the solution was designed for (8), is relatively low (9, 10). The relatively small number of loci that can be simultaneously analyzed by PCR-based methods limits the opportunities to account for tissue of origin and other sample-specific factors. In addition, common population polymorphisms within homopolymers can reduce the sensitivity of PCR-based MSI detection methods, especially when matched normal samples are unavailable (11, 12).

Next-Generation Sequencing (NGS) based MSI detection allows the simultaneous analysis of a larger number of microsatellite regions, thus limiting the impact of sample-specific factors, including tissue of origin or population-specific variation in microsatellite length (13). In addition, NGS-based MSI analysis can be combined with other cancer-related molecular signatures and genetic lesions, facilitating the adoption of MSI clinical testing and increasing the number of patients considered for immunotherapy (14). Indeed NGS-based methods that rely on analysis of paired tumor-normal samples support accurate MSI detection across multiple tumor types (15). However, this data type is not commonly available in the clinic. Whereas NGS-based methods that leverage information from tumor-only data would circumvent this challenge, inter- and intra-tumor specific differences in the frequency and position of MSI diagnostic events (16) (17, 18) still impact their accuracy (15). For example, many MSI events are private to one sample, and frequently occurring events can be tumor-type specific (16). Additionally, microsatellite regions are often polymorphic in healthy individuals, and their sequence differs across the human population (7, 19). All these factors limit the analytical performance of methods that rely on a baseline reference distribution to determine MSI status.

To address these limitations, we developed MSIdetect, a new MSI detection method. MSIdetect uses a curve fitting algorithm, thus accounting for the impact of tumor content and indel burden on homopolymer instability. To minimize the effect of intra- and inter- tumor-specific factors, we additionally restrict our analysis to a set of ~100 homopolymer regions that we found are minimally variable between tissues and individuals. Using a large cohort of clinical samples, we demonstrate that MSIdetect can sensitively detect MSI signatures from tumor-only data in various cancer types, even in samples with limited tumor content.

## Results and discussion

### NGS-based detection of MSI using Whole Exome Sequencing data

**Figure 1:**
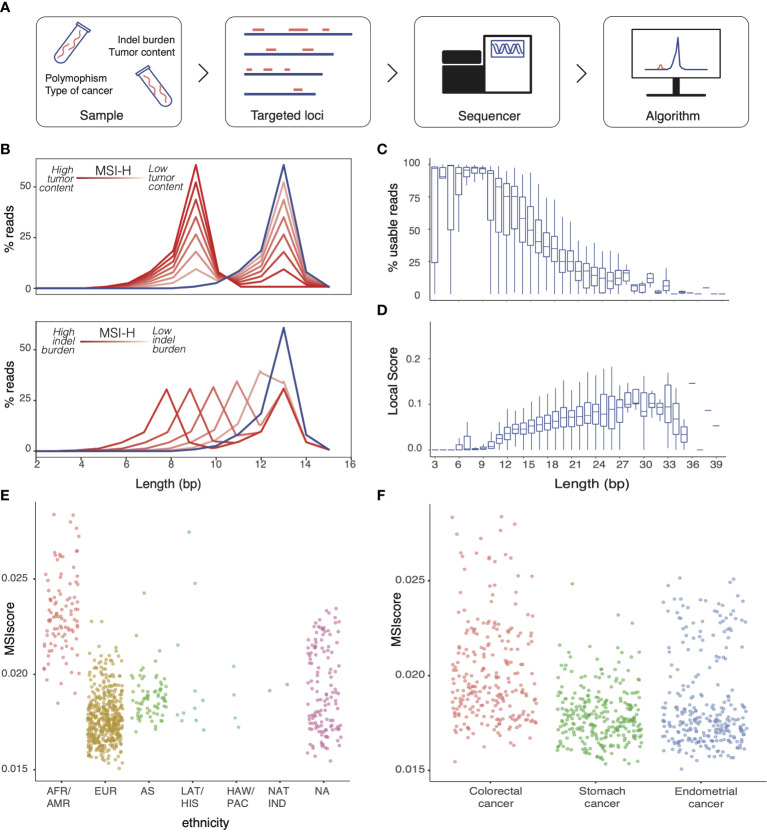
Factors limiting MSI detection in NGS workflow **(A)** Factors impacting detection of MSI in tumor-only NGS workflows **(B)** Schematic representation of the impact of increased indel burden (bottom panel) and tumor content (top panel) on the homopolymer length distribution measured by NGS at an illustrative homopolymer (MSI-H, red). Line color darkness correlates with decreased tumor content (top panel) or indel burden (bottom panel). Reference homopolymer length distribution for microsatellite stable is depicted in blue. Distribution of **(C)** Fraction of usable reads per total number of reads mapping to the homopolymer and **(D)** homopolymer score for homopolymers of the same length. MSI score obtained with MSIdetect using WES homopolymers for microsatellite stable (MSS) samples derived from **(E)** individuals of different ethnic origin and for samples from **(F)** different tumor types.

MMR deficiency (dMMR) results in microsatellite contraction and expansion. To optimize detection of this signature using NGS from tumor-only data, MSI detection solutions must account for the factors that can limit their sensitivity and specificity (**Figure 1A**). In NGS workflows, microsatellite instability is reflected by a difference, relative to a normal reference, in the distribution of read counts supporting different microsatellite lengths. MSIdetect relies on a curve-fitting algorithm (described in Materials and Methods section) that accounts for the impact of tumor heterogeneity and the indel burden on microsatellite length distribution (**Figure 1B**).

We used publicly available The Cancer Genome Atlas (TCGA) Whole Exome Sequencing (WES) data from 363 Colorectal Adenocarcinoma, 428 Stomach Adenocarcinoma and 492 Uterine Corpus Endometrial Carcinoma samples, with known MSI status (20), to investigate how different limiting factors (**Figure 1A**) might contribute to miscalls in our analytical workflows. Homopolymer length impact MSI detection by NGS in two ways. First, homopolymer length negatively correlates with the fraction of reads that span the entirety of the region, and that can be used by the algorithm to infer the region’s length stability (**Figure 1C**). In addition, the length distribution of relatively short homopolymers is very stable even in MSI-H samples, limiting their value to measure local instability (**Figure 1D**). These two factors are likely to define an optimal range of homopolymer length for MSI detection by NGS-based approaches.

In addition to indel burden and tumor content that is accounted for by the algorithm, other samples characteristics can also impact results. Specifically, homopolymers replication is error-prone (21), with MMR independent factors such as ethnicity (**Figure 1E**) or tissue origin (**Figure 1F**) impacting homopolymer length, as reflected by changes in MSIscore, in MSS samples.

### Identification of homopolymers for optimal NGS-based detection of MSI

**Figure 2:**
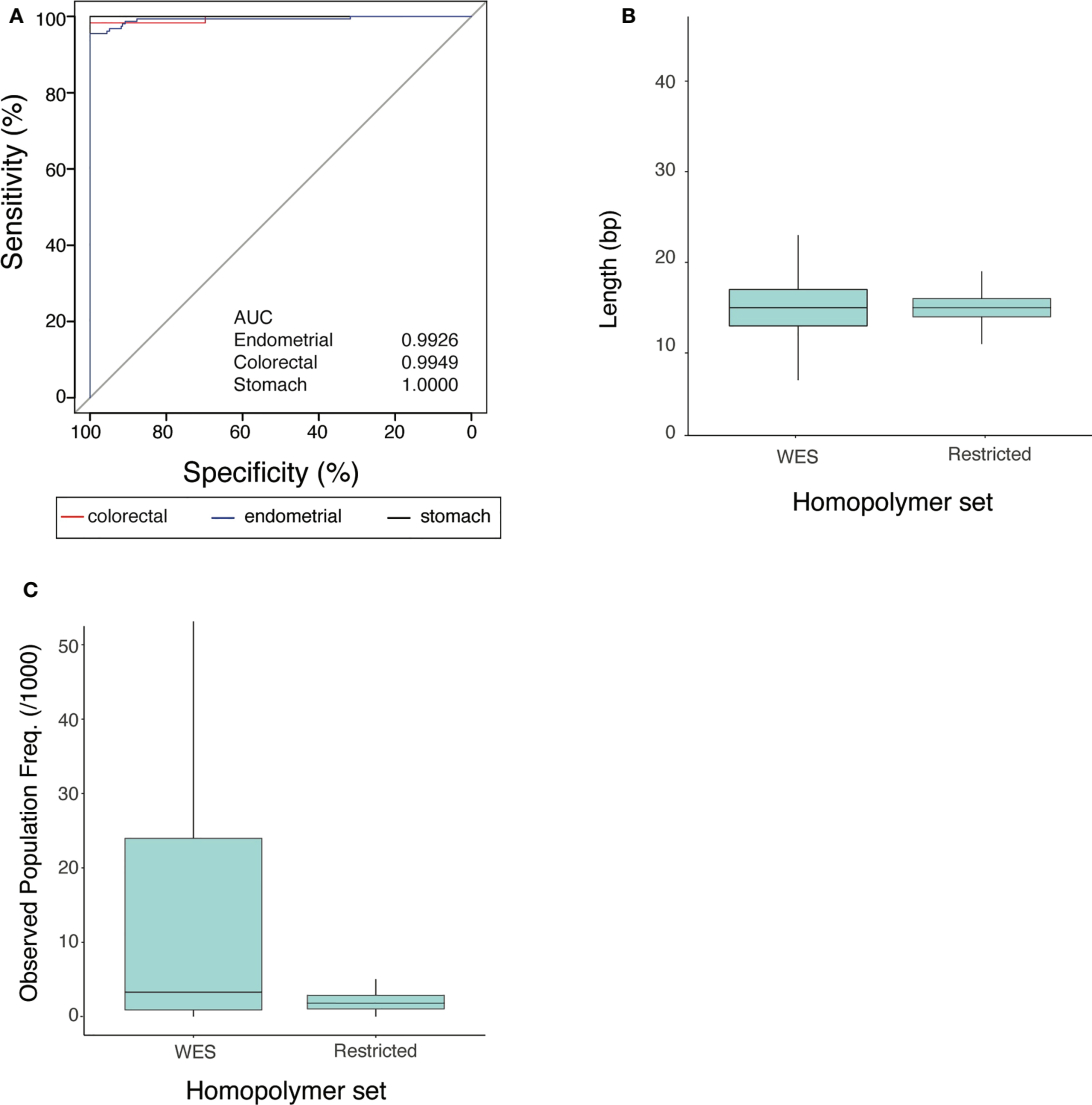
Properties of the MSIdetect restricted homopolymer set **(A)** Receiver Operating curves and corresponding Area Under the Curve (AUC) values (in the inset) for endometrial (blue), colorectal (red) and stomach (black) cancers for MSI classification by MSIdetect using WES homopolymers given the MSI status reported by TCGA. **(B)** Homopolymer length distribution in WES and in the restricted set. **(C)** Distribution of average variant population frequency observed in gnomAD for homopolymers in WES and in the restricted set with lengths ranging from 11-25 bp.

We computed the MSI score based on all homopolymers captured in the WES datasets (3602 loci (22), **Supplementary Figure 1A**) and assessed the concordance between MSIdetect results and pre-determined MSI status. We plotted the true-positive rate as a function of the false-negative rate obtained for the different tissues (**Figure 2A**). We found that MSIdetect results were highly concordant with MSI status (AUC>0.9926). When all homopolymers captured by the WES data set are considered, we observed tissue-specific differences in accuracy, with results being less accurate in Uterine Corpus Endometrial Carcinoma (AUC=0.9926), followed by Colorectal Adenocarcinoma (AUC=0.9976) and Stomach Adenocarcinoma (AUC=1.000).

We compared the analytical performance of MSIdetect with that of two other widely used NGS-based MSI detection algorithms mSINGS (22) and MANTIS (15). These algorithms were chosen because, like MSIdetect, they rely on comparing microsatellite allele length distributions. Similar to MSIdetect, mSINGS (22) is compatible with tumor-only data, whereas MANTIS (15) relies on comparing the results obtained for a tumor sample with its matched normal sample. Like MSIdetect, the other algorithms are less accurate in endometrial cancer, followed by colorectal and stomach cancer (**Supplementary Figures 1B-D**). In all cancer types considered, MANTIS showed the highest overall performance with >97.4% sensitivity at 95% specificity (**Table 1**). We attribute the higher analytical performance of this algorithm to the limited impact of sample-specific factors (**Figure 1A**) on the results of approaches such as MANTIS (13, 15) that rely on comparison to matched normal samples. Between the two algorithms that rely on comparison to a set of baseline samples, MSIdetect had a slightly higher performance with >96.1% compared to >94.9% sensitivity for mSINGS at a 95% specificity.

**Table 1:** Sensitivity at 95% specificity for different algorithms in endometrial, colorectal and stomach cancer using WES homopolymers.

	Endometrial	Colorectal	Stomach
MSIdetect*	96.1% [98.6-91.8]	98.3% [99.9-90.9]	100.0% [100-95.7]
mSINGS*	94.9% [97.8-90.1]	98.3% [99.9-90.9]	100.0% [100-95.7]
MANTIS**	97.4% [99.3-93.6]	98.1% [99.9-90.1]	100.0% [100-95.7]

Asterisks indicate that * algorithm relies on comparison of tumor sample with a set of baseline samples or ** matching normal sample. Values inside square brackets indicate the 95% Confidence Interval for all estimates.

We hypothesized that homopolymer selection could account, at least in part, for some of the limitations of MSI detection solutions that rely on NGS-based approaches, particularly those that leverage information from tumor-only data. This hypothesis is supported by evidence that the size and composition of the set of homopolymers considered impacts analytical performance (15).

To identify a set of homopolymers that would optimize MSI detection by NGS, we considered half of the samples in the pan-cancer dataset, hereafter referred to as training set, to identify homopolymers that would maximize the differences between MSI-H and MSS samples across multiple tumor types. To do so, we estimated the score at all homopolymers using MSIdetect. We defined groups of homopolymers based on whether the score in samples classified as MSI-H was higher than a fixed percentile (between 25-95%) of the maximal score observed for that homopolymer in samples classified as MSS from the same cancer type (**Supplementary Table S1**). Based on the MSIscore we computed for samples in the training set using the different homopolymers combinations (**Supplementary Figures 2A-C**) we determined the analytical performance and MSIscore variability associated with the different homopolymer sets. Based on these results (**Supplementary Table S2**) we concluded that the 136 homopolymers with a score in MSI-H samples higher than MSS in samples in than 80% of samples, offers optimal MSI detection relative to the other tested homopolymer sets. We hereafter refer to this homopolymer set as restricted homopolymer set.

We investigated what distinguished homopolymers in the restricted set from the remaining homopolymers captured by the WES solution. Relative to all considered homopolymers, those in the restricted set tend to be of intermediate length (median 15 bp, 11-25 bp, **Figure 2B**). This intermediate length is likely to facilitate read mapping and render homopolymers sensitive to dMMR dependent expansion and contraction.

In addition, we found that homopolymers in the restricted set have ~1.8x lower average population frequency amongst humans, based on gnomAD (two-tailed, Mann-Whitney test p-value<0.0002, **Figure 2C**) than other homopolymers of the same length (11-25 nt) which is likely to minimize the impact of population polymorphism in MSI score.

To assess the impact of implementing analysis of the restricted set on MSIdetect’s analytical performance we considered the remaining samples of the pan-cancer data set, hereafter referred to as the test set. Restricting MSIdetect analysis to the restricted set of homopolymers improves performance relative to when all homopolymers in WES are considered. Specifically, restricting the analysis to the restricted homopolymer is associated with 100% sensitivity at 95% specificity (**Table 2**) and an increase in AUC (>0.995) in all tested tissues (**Supplementary Table S3**). This difference is also reflected in a slight increase in AUC (0.9995 for restricted homopolymer set compared to 0.9926 for all homopolymers). Like MSIdetect, the performance of the other algorithms tested (**Table 2**; **Supplementary Table S3**) also improved when only the restricted homopolymer set was considered. In line with previous work (15), this observation supports the use of specific microsatellite marker, including the set identified here, can improve the analytical performance of NGS-based methods of MSI detection.

**Table 2:** Sensitivity at 95% specificity for different algorithms when considering restricted homopolymer set in endometrial, colorectal and stomach cancer.

	Endometrial	Colorectal	Stomach
MSIdetect*	100% [100-95.8]	100% [100-87.6]	100% [100-91-9]
mSINGS*	98.8% [100-93.7]	100% [100-87.6]	100% [100-91.9]
MANTIS**	100% [100-95.8]	100% [100-86.3]	100% [100-91.9]

Asterisks indicate that * algorithm relies on comparison of tumor sample with a set of baseline samples or ** matching normal sample. Values inside square brackets indicate the 95% Confidence Interval for all estimates.

In conclusion, the increase in analytical performance associated with the combination of algorithm and restricted set homopolymer regions limits the impact of biological and technical factors on the ability to detect by NGS the differences in homopolymer length distribution caused by loss of MMR gene function, using tumor-only data.

### MSIdetect is sensitive and specific in colorectal and endometrial cancer

Next, we sought to assess the analytical performance of MSIdetect in combination with the restricted homopolymer set in Formalin-Fixed Paraffin-Embedded (FFPE) clinical samples.

**Figure 3:**
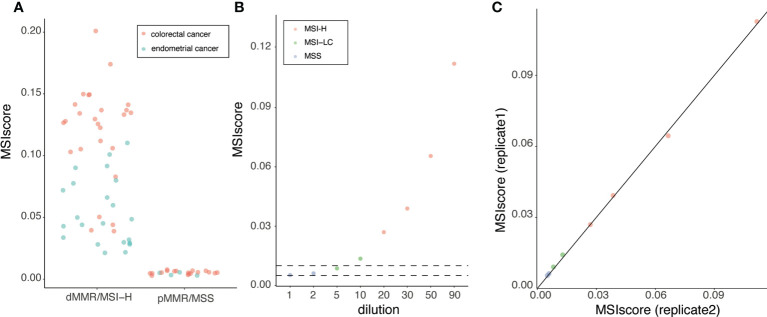
MSI detection in colorectal and endometrial FFPE clinical samples **(A)** MSIscore obtained for colorectal or endometrial cancer samples. Samples were grouped based on their respective MSI-PCR and IHC result. Each point corresponds to one sample colored by tissue of origin (refer to legend in figure) **(B)** MSIscore for a dilution series containing between 1 and 90% (x-axis) of DNA extracted from one MSI-H tumor DNA diluted in MSS tumor DNA in duplicates. Each point corresponds to one sample. Samples are colored according to results of MSI-PCR test (refer to legend in figure). **(C)** MSIscore obtained for replicate 1 and 2 for dilution series of MSI-H DNA in MSS DNA.

We first considered colorectal and endometrial cancer samples (44 and 30 samples, respectively) with MMR and MSI status defined using immunohistochemistry (IHC) and PCR (MSI-PCR) methods, respectively. The MSI and MMR status for these samples were concordant (**Supplementary Table S4**). We generated NGS data for homopolymers in the restricted set for these samples. We observe no overlap between the distribution of score obtained using MSIdetect for these samples dMMR/MSI-H from pMMR/MSS samples indicating the method allows distinction of. he two classes with 100% sensitivity and specificity (**Figure 3A**). To define the MSIscore threshold, we considered the standard deviation and the median score estimated for MSS samples (0.001 and 0.0028). We defined the thresholds for sample classification as follows: MSS samples have an MSIscore smaller than 0.005; MSI low confidence (MSI-LC) an MSIscore between 0.005 and 0.01; and MSI High confidence (MSI-H) an MSIscore higher than 0.010. These thresholds were chosen to maximize MSIdetect analytical performance. Change in the number or composition of homopolymer set considered should entail reevaluation of these thresholds (Supplementary Note 1).

To investigate the impact of tumor content on MSI detection performance, we diluted (1-90%), in replicate, one MSI-H tumor DNA in MSS tumor DNA from samples with relatively high tumor content samples. As expected, the MSIscore decreased with decreasing amounts of MSI-H tumor DNA (**Figure 3B**). The impact on sample classification of this decrease is similar to what was seen for MSI-PCR (**Figure 3B**). MSIscore is highly correlated between replicates (R>0.99, p-value<2X10^-8^, **Figure 3C**), supporting the robustness of the approach. MSIdetect classified dilutions with limited MSI tumor DNA content (<2%) as MSI-LC indicating that MSIscore is sensitive to relatively low levels of homopolymer instability.

### MSIdetect detects MMR deficiency in various cancers, including glioma and sebaceous adenomas and carcinomas

**Figure 4:**
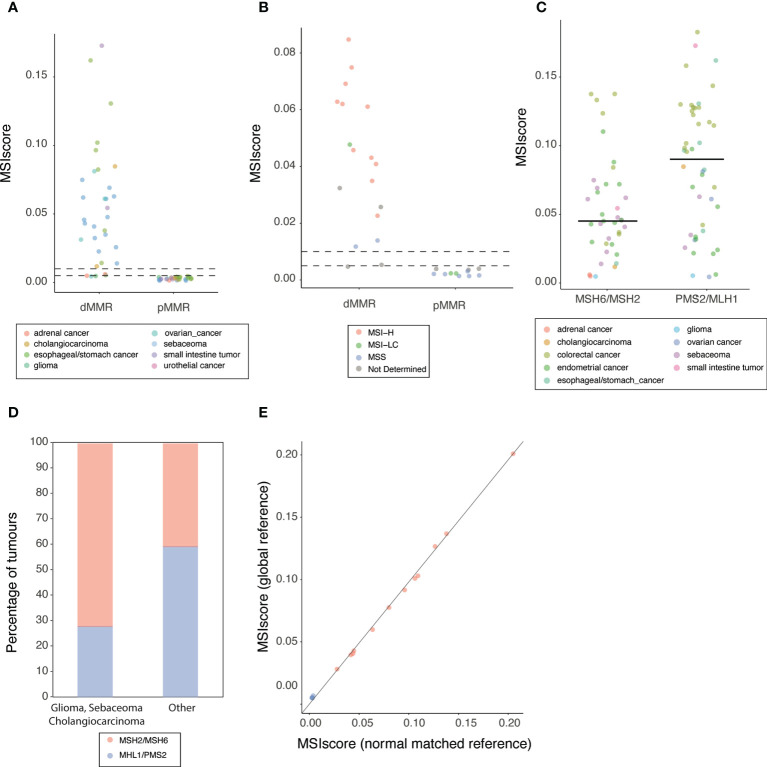
MSI detection in FFPE clinical samples **(A)** MSIscore obtained for dMMR or pMMR FFPE samples. Each point corresponds to one sample colored by tissue of origin (refer to legend in figure). Horizontal lines top to bottom indicates MSI-HC and MSI-LC threshold respectively **(B)** MSIscore obtained for glioma, sebaceoma and cholangiocarcinoma FFPE samples classified by IHC as dMMR or pMMR. Each point corresponds to one sample colored by MSI-PCR status. Horizontal lines top to bottom indicates MSI-H and MSI-LC threshold respectively. **(C)** MSIscore obtained for dMMR FFPE samples grouped by pairs of protein lost (x-axis) Each point corresponds to one sample colored by tissue of origin (refer to legend in figure). Horizontal lines indicate the median score for the group. **(D)** Histogram of the percentage of genes with detected loss of MSH2/MSH6 or MHL1/PSM2 grouped by cancer type **(E)** MSIscore obtained using either a global reference (y-axis) or a reference build using a matched-normal samples. Each point corresponds to one sample colored by MSI-PCR result.

Next, we considered samples from tumor types where MSI detection is more challenging, including glioma. When we considered the MMR status based on IHC, the method of preference for classification of these samples, we found that MSIdetect is 100% specific and 91% sensitive (**Figure 4A**) when only challenging samples are included. For 2 out of the 3 dMMR samples missed by MSIdetect (**Figure 4A**), MSI-PCR results were also available (**Supplementary Table S4**). In both cases, the number of loci found to be unstable (2/5) was low and below the recommended test’s threshold for MSI classification. The remaining sample was from glioma, where MSI-PCR is not routinely performed due to the lack of sensitivity of MSI-H status detection in this tumor type.

In addition to glioma, MSI detection is also challenging in other tumors such as cholangiocarcinoma, urothelial or adrenal carcinoma and sebaceous adenoma or carcinoma (23–26). When we considered these 3 cancers, we found that 94% of the 18 dMMR samples from these cancer types were classified as MSI by MSI detect. This includes 2 samples classified by MSI-PCR as MSS, 1 sebaceoma and 1 cholangiocarcinoma (**Figure 4B**).

Differences in mutational patterns between tumor types have been proposed to account for decreased MSI detection sensitivity (27, 28). Given the relatively small number of samples were expression of only one protein in the functional heterodimer pairs MLH1/PMS2 or MSH2/MSH6 is loss (**Supplementary Table S4**) we grouped samples according to heterodimer loss of function.

Interestingly, dMMR samples where MLH1 or PMS2 (median MSIscore=0.090) were lost have significantly higher levels of microsatellite instability (two-tailed Mann-Whitney test p-value<0.005) than dMMR samples with loss of function in MSH2 or MSH6 (median MSIscore=0.045) (**Figure 4C**). Loss of MSH6 function is known to result in lower levels of microsatellite instability (29). However the relatively low number of samples where only MSH2 or MSH6 appears to be lost by IHC, that we attribute to protein regulation by dimer stabilization (30), limits our ability to assess the impact of loss of function either gene to the MSIscore observed of MHS2/MHS6 deficient tumors.

This difference in MSIscore observed between MLH1/PMS2 and MHS2/MHS6 deficient tumors explains, at least in part, the low levels of instability observed in glioma and sebaceous adenoma or carcinoma. Indeed, in these tumors, MSH2/MSH6 mutations are significantly (two-tailed Fisher’s exact test p-value<0.005) more frequent (13/18 cases) than in the rest of the cohort where MSH2/MSH6 mutations are less common (26/64 samples) (**Figure 4D**).

For a subset, in addition to tumor samples, non-tumor matched normal material was also available (16 samples). These samples allowed us to assess the impact of the results obtained when microsatellite instability is measured relative to a panel of normal samples (global reference) or a matched normal sample (Methods). We observed a strong correlation (R= 0.996, correlation test p-value<2.2X10^-16^, **Figure 4E**) between the MSIscore obtained using the global and match reference. The observation that the score is similar when using global or normal matched supports that the combination of algorithm and restricted homopolymer set allows overcoming some of the challenges of tumor only analysis of microsatellite regions.

## Conclusion

Mismatch repair deficiency (dMMR) confers sensitivity to immune checkpoint inhibition therapy across different cancer types (3–5). However, and despite its pan-cancer value, clinical detection of this molecular signatures is often restricted to colorectal and endometrial cancer where this molecular phenotype is most common (20). This is in part because dedicated assays, analysis of protein loss of function by immunohistochemistry or of MSI by PCR, are still preferred to next generation sequencing (NGS) based methods (6) but require tumors to be matched to paired normal samples for analysis of non-colorectal cancers sample. The main advantage of NGS based methods is that they allow integration of MSI detection as part of comprehensive molecular profiling assays, supporting adoption of dMMR testing and increasing the number of patients considered for immunotherapy (19).

Here we describe MSIdetect, a NGS based solution developed to support accurate detection of MSI from tumor-only data. We identified the sample-specific and analytical factors that limit performance MSI detection by NGS. We found that when considering tumor only data, accounting for homopolymer properties, indel burden and tumor content increases sensitivity. However, algorithm improvements alone cannot account for the impact of tissue of origin and patient ethnicity when only tumor samples are available. To address this limitation, we used publicly available data to identify a set of loci that is minimally impacted by sample specific factors. Integration of these insights limits the impact of the identified confounders on the results from tumor-only data and supports performances comparable to what can be obtained when normal matched samples are available.

We investigated the accuracy of MSIdetect in a diverse cohort of clinical samples using results of IHC as ground truth. As highlighted by a recent meta-analysis the evidence supporting the value of MSI-NGS solutions in non-colorectal cancers is low, demonstrating the need for development and validation of NGS based methods that can accurately detect MSI in other cancer types (31).

We show that MSIdetect is 100% accurate in colorectal and endometrial cancer. This is despite the MSIscore being lower in endometrial relative to colorectal cancer, consistent with the previously reported (17, 18) differences in size and frequency of indels at microsatellites in these two cancer types. Tissue specific differences on the impact of loss of MMR on microsatellite instability have also been reported in other cancer types, including glioma or sebaceous adenoma or carcinoma, where MSI detection is known to be challenging (23, 24, 26, 27). When MSIdetect was used to analyze samples from these cancer types we observed a slightly lower overall accuracy (accuracy 97.8%). For 2 out of the 3 false negative samples, MSI status based on a commonly used PCR based method was also available. Both these samples were also classified by the PCR based method as MSS indicating that the impact of loss of MMR function on expansion and contraction of homopolymer is low and generally hard to detect in these cases. Interestingly, we found that dMMR in these samples is caused by loss of MSH6 alone or together with MSH2 which is associated with loss of sensitivity to detect MSI (27, 28).

In summary, we show that MSIdetect supports accurate detection of MSI signatures in different cancer types. Its adoption alone or as part of molecular profiling solutions can increase the number of patients identified that are likely to benefit from immune checkpoint inhibitor therapy, particularly in cancers where PCR based MSI detection methods were found to have limited sensitivity and in samples with low tumor content.

## Materials and methods

### MSI analysis of public data

Tumor-normal whole-exome sequencing data for 78, 85 and 156 MSI-H and 245, 265 and 274 MSS colorectal adenocarcinoma, stomach adenocarcinoma, and uterine endometrial carcinoma, respectively, was obtained from The Cancer Genome Atlas (TCGA). Aligned BAM files (to hg38) and associated clinical information for all the samples was downloaded from Genomic Data Commons.

We considered the coverage by sufficient quality reads and excluded from our analysis homopolymers with insufficient coverage.

### Description and MSI calling using MSIdetect

MSIdetect score for sample *j* is calculated as the median homopolymer score, *HPscore*, for all homopolymers considered in the analysis. The *HPscore* for homopolymer *i* in sample *j* is defined as the product between the values of the parameters, *p1* and *p2*, that maximize the fit between the read length distribution obtained for homopolymer *i* in sample *j* $\left(D_{j}^{i}\right)$ with the read length distribution of homopolymer *i* in reference MSS sample(s), hereafter referred to as reference $\left(D_{ref}^{i}\right)$ , using the multiparametric function defined by equation 1.

equation 1


$$(p_{1},\;p_{2},\;p_{3})\;=\;\text{arg}\;\text{min}(\int_{0}^{\int_{\max}}{|D}_{j}^{i}(l)-T(D_{\mathrm{ref}}^{i}(l),p_{1},p_{2},p_{3})|\mathrm{dl})$$

Where *l_max_* is the maximum homopolymer length observed in $D_{ref}^{i},\;\;l$ is the homopolymer length and T is the function which transform $D_{ref}^{i}\;(\;l)$ according to the transformation described below (equation 2):

equation 2


$$T(D_{ref}^{i}(l),p_{1},p_{2},p_{3})\;=\;(1-p_{1})\;\cdot \;p_{3}\;\cdot \;D_{ref}^{i}((\frac{\;l-l_{ref}}{p_{3}}\;+\;l_{ref})\;-\;p_{2}\;\cdot \;l_{ref})+\;p_{1}\;\cdot \;D_{ref}^{i}(l)$$

where *l_ref_* is the reference length at this locus.

For a given homopolymer *i*, *p1* is the difference between the measured height of the read distribution peak in sample *j* and in the reference distribution; *p2* is the maximum difference observed in homopolymer length between sample *j* and the reference and reflects the difference in peak position in sample *j* relative to the reference distribution; and p3 is the width of the length distribution for homopolymer *i* in sample *j*. As depicted in **Figure 1B**, p1 and p2 are expected to change as function of tumor content and indel burden, respectively. The parameter p3 captures changes in homopolymer lengths distribution width between the sample and the reference distribution.

Because in MSS samples the value of either *p1* or *p2* will be close to 0, meaning that value taken by any of the other parameters on score, we chosen to consider only *p1* and *p2* in the estimation of the homopolymer score.

Reference length distribution is pre-computed from aligned sequence data for MSS or matched normal samples. Unless stated otherwise analysis of TGCA and clinical samples were done based on the comparison to a reference length distribution computed using aligned sequencing data for 10 MSS samples selected randomly from either the cancer genome atlas (TCGA) or clinical samples, respectively. As documented, in Supplementary Note 2 the set of MSS samples chosen to build the reference distribution minimally impacts *MSIscore*.

Only reads that are perfectly matched to the homopolymer region excluding the homopolymer region plus or minus 3 nucleotides were considered. Reads mapping to the forward and reverse strand are considered separately and $HP\;score_{j}^{i}$ is the average of the score in both directions.

### MSI calling using mSINGS

We considered 25 MSS samples from colorectal adenocarcinoma, stomach adenocarcinoma and uterine endometrial carcinoma to build the reference distribution using default parameters. Loci with no variance were excluded as recommended by the developers. MSI score was computed as described by developer’s version v.4.0.

### MSI calling using MANTIS

MSI score was computed using MANTIS (version v1.0.5) and the parameters recommended in (15), (mrq = 20, mlq = 25,mlc = 20,mrr = 1) for tumor and normal matched paired samples.

### Analysis of human polymorphism

We extracted variants reported in from gnomAD v2.1.1 that impact homopolymer length distribution and computed their frequency using their allele count across populations.

### Characterization of clinical samples

Tissue samples from patients diagnosed for their MSI and MMR status between 2016 and 2020 in the pathology department of the *Hospices Civils de Lyon* (HCL, France). The properties of the clinical samples are listed in **Supplementary Table S4**. Non-CRC carcinomas were classified according to the World Health Organization (WHO) histopathological classifications and were reviewed independently by two pathologists for tumor classification and cellularity. MSI status was done using multiplex PCR and capillary electrophoresis-based assay PCR– based MSI test used in our laboratory was done accordingly to the instructions provided by the manufacturers (Promega Corporation, Madison, WI, USA). Two µL of DNA which concentration was adjusted to 10 ng/µL was used to co-amplify by multiplex PCR 5 mononucleotide repeat markers: BAT-25, BAT-26, NR-21, NR-24 and MONO-27, and 2 pentanucleotide repeat markers (Penta C and Penta D). The PCR products are separated by capillary electrophoresis using an Applied Biosystems^®^ 3130 Genetic Analyzer. The output data were analyzed with GeneMapper^®^ software (Applied Biosystems) to determine MSI status of test samples.

To investigate the mismatch repair protein (MMR) expression standard 4-µm thick FFPE tumor sections were subjected to immunohistochemistry staining (IHC) analysis using MLH1 antibody (Ab) (clone G168-728, Ventana Ab, 1/25), MSH2 Ab (clone 25D12 DBS Clinisciences, 1/25), MSH6 Ab (clone 44 BD Biosciences, 1/500) and PMS2 Ab (A16-4, Pharmingen, 1/200) on a Ventana automated staining platform (BenchMark ULTRA, Tucson, AZ, USA). Internal positive control was included in the tissue section. Loss of MMR expression was considered in case of total absence of nuclear expression by tumor cells while normal cells express the protein (32–34). All samples were from the tumor bank “Tissu-tumorotheque Est” and “Tissu-tumorotheque Sud” of the Biological Resource Centre (Centre de Ressource Biologique, CRB) of the HCL (Lyon, France).

### Clinical sample preparation and sequencing

The regions corresponding to the restricted homopolymer set (136 loci) plus their neighboring genomic regions in hg19 were downloaded and DNA repeat content analyzed. After exclusion of homopolymers within repetitive regions probes of 117 homopolymers were designed and ordered.

Targeted libraries were created using capture-based enrichment technology. First, 50 ng of input FFPE extracted genomic DNA was enzymatically fragmented, end-repaired and A-tailed, followed by ligation to custom short y-shaped adapters. The ligation products were purified with AMPure beads (Beckman Coulter) and then amplified by PCR for 10 to 14 cycles (depending on the amount of input DNA) using Illumina-compatible primers with dual-indices. Amplified libraries were cleaned-up with AMPure beads (Beckman Coulter) and libraries pooled to give a total of 1.8 µg. The pools were mixed with human Cot-1 DNA (Life Technologies) and xGen Universal Blockers-TS Mix oligos (Integrated DNA Technologies) and lyophilized. Pellets were resuspended in a hybridization mixture, denatured for 10 min at 95°C and incubated for 4-16 h at 65°C in the presence of biotinylated probes (xGEN Lockdown IDT^®^). Probe-hybridized library fragments were captured with Dynabeads M270 Streptavidin (Invitrogen) and then washed. The captured libraries were amplified by PCR for 15 cycles and cleaned-up using AMPure beads (Beckman Coulter).

Paired end (150 base pair) reads libraries were sequenced on the Illumina Miseq or NextSeq platform (Illumina Inc., San Diego, CA, USA). Sequencing data was processed using the SOPHiA GENETICS proprietary pipelines accessible through SOPHiA GENETICS DDM platform. All samples were sequenced to approximately 1000 x coverage which is more than the estimated minimal depth required to ensure accurate distinction between MSI and MSS samples (Supplementary Note 3).

### Statistical analysis

Statistical analysis and graphics were done using R.

## Data availability statement

MSIdetect algorithm is a SOPHiA GENETICS proprietary algorithm and is available as part of SOPHiA GENETICSDDM platform. The original contributions presented in the study are included in the article/**Supplementary Material**. Further inquiries can be directed to the corresponding author.

## Ethics statement

The studies involving human participants were reviewed and approved by Biological Resource Center of the Hospices Civils de Lyon. The patients/participants provided their written informed consent to participate in this study. Written informed consent was obtained from the individual(s) for the publication of any potentially identifiable images or data included in this article.

## Author contributions

AM, FM, LS, XX, AW, and ZX conceived and planned the study. FM and LS developed MSIdetect pipeline. ES and GF generated NGS data under AW supervision, FM analyzed NGS dataset with support from MW and XX. CF-P, TF, VH, BBal, MD, BBan, DM, and JL were responsible for sample collection. CC, MB, and PM coordinated the study, AM and FM did the statistical analysis and prepared figures and tables. CF-P, JL, and ZX provided intellectual input for data interpretation. AM wrote the first draft of the manuscript. All authors reviewed and approved the final manuscript.

## Acknowledgments

We thank Corinne Perrin and Elisabeth Blasco from the tumour bank “Tissu-tumorotheque Est” and “Tissu-tumorotheque Sud” of the Hospices Civils de Lyon’s Biological Resource Centre for the collection of patient’s consents. We thank Aurélie Gauthier (GHE pathology department) for its technical assistance to handle and send the MMR samples, Dr PP Bringuier and M Barritault who facilitated the sending of the samples.

## Conflict of interest

AM, FM, LS, ES, GF, AW, MW, XX, CC, MB, PM, and ZX are SOPHiA GENETICS employees. CF-P reports sponsorship for meeting attendance from Roche and personal fees for advisory board work from Novartis, outside the submitted work. JL reports consulting for SOPHiA GENETICS and Decibio and personal fees for advisory board work and attendance to scientific meeting by Roche, Astra-Zeneca, BMS, Lilly and Nanostring.

The remaining authors declare that the research was conducted in the absence of any commercial or financial relationships that could be construed as a potential conflict of interest.

## Publisher’s note

All claims expressed in this article are solely those of the authors and do not necessarily represent those of their affiliated organizations, or those of the publisher, the editors and the reviewers. Any product that may be evaluated in this article, or claim that may be made by its manufacturer, is not guaranteed or endorsed by the publisher.

## Supplementary material

Supplementary Table 4 Properties of the clinical samples used in the study.

Supplementary Figure 1
**(A)** Score obtained with MSIdetect, using WES homopolymers in colorectal, stomach and endometrial cancer. Each point corresponds to one sample colored by reported MSI status (refer to legend in the figure). Receiver Operating curves and corresponding Area Under the Curve (AUC) values (in the inset) for endometrial **(B)**, colorectal **(C)** and stomach **(D)** cancers for MSI classification by MSIdetect, mSIGNS and MANTIS using WES homopolymers given the MSI status reported by TCGA.

Supplementary Figure 2 Distribution of the MSIscore for microsatellite instability high (MSI-H, red) and stable (MSS, blue) samples in the training set using the different homopolymers combinations in endometrial **(A)**, colorectal **(B)** and Stomach **(C)** cancer.

## References

[1] Kunkel TA. Evolving views of DNA replication (in)fidelity. Cold Spring Harb Symp Quant Biol (2009) 74:91–101. doi: 10.1101/sqb.2009.74.027 19903750 PMC3628614

[2] Hsieh P Yamane K. DNA Mismatch repair: molecular mechanism, cancer, and ageing. Mech Ageing Dev (2008) 129:391–407. doi: 10.1016/j.mad.2008.02.012 18406444 PMC2574955

[3] Le DT Uram JN Wang H Bartlett BR Kemberling H Eyring AD. PD-1 blockade in tumors with mismatch-repair deficiency. N Engl J Med (2015) 372:2509–20. doi: 10.1056/NEJMoa1500596 PMC4481136 26028255

[4] Le DT Durham JN Smith KN Wang H Bartlett BR Aulakh LK. Mismatch repair deficiency predicts response of solid tumors to PD-1 blockade. Science (2017) 357:409–13. doi: 10.1126/science.aan6733 PMC5576142 28596308

[5] Marcus L Lemery SJ Keegan P Pazdur R. FDA Approval summary: Pembrolizumab for the treatment of microsatellite instability-high solid tumors. Clin Cancer Res (2019) 25:3753–8. doi: 10.1158/1078-0432.CCR-18-4070 30787022

[6] Luchini C Bibeau F Ligtenberg MJL Singh N Nottegar A Bosse T. ESMO recommendations on microsatellite instability testing for immunotherapy in cancer, and its relationship with PD-1/PD-L1 expression and tumour mutational burden: a systematic review-based approach. Ann Oncol (2019) 30:1232–43. doi: 10.1093/annonc/mdz116 31056702

[7] Ellegren H. Microsatellites: simple sequences with complex evolution. Nat Rev Genet (2004) 5:435–45. doi: 10.1038/nrg1348 15153996

[8] Goel A Nagasaka T Hamelin R Boland CR. An optimized pentaplex PCR for detecting DNA mismatch repair-deficient colorectal cancers. PloS One (2010) 5:e9393. doi: 10.1371/journal.pone.0009393 20195377 PMC2827558

[9] Stelloo E Jansen AML Osse EM Nout RA Creutzberg CL Ruano D. Practical guidance for mismatch repair-deficiency testing in endometrial cancer. Ann Oncol (2017) 28:96–102. doi: 10.1093/annonc/mdw542 27742654

[10] Siemanowski J Schömig-Markiefka B Buhl T Haak A Siebolts U Dietmaier W. Managing difficulties of microsatellite instability testing in endometrial cancer-limitations and advantages of four different PCR-based approaches. Cancers (Basel) (2021) 13:1268. doi: 10.3390/cancers13061268 33809329 PMC8000432

[11] Buhard O Cattaneo F Wong YF Yim SF Friedman E Flejou J-F. Multipopulation analysis of polymorphisms in five mononucleotide repeats used to determine the microsatellite instability status of human tumors. J Clin Oncol (2006) 24:241–51. doi: 10.1200/JCO.2005.02.7227 16330668

[12] Campanella NC Berardinelli GN Scapulatempo-Neto C Viana D Palmero EI Pereira R. Optimization of a pentaplex panel for MSI analysis without control DNA in a Brazilian population: correlation with ancestry markers. Eur J Hum Genet (2014) 22:875–80. doi: 10.1038/ejhg.2013.256 PMC4060109 24193342

[13] Baudrin LG Deleuze J-F How-Kit A. Molecular and computational methods for the detection of microsatellite instability in cancer. Front Oncol (2018) 8:621. doi: 10.3389/fonc.2018.00621 30631754 PMC6315116

[14] Albayrak A Garrido-Castro AC Giannakis M Umeton R Manam MD Stover EH. Clinical pan-cancer assessment of mismatch repair deficiency using tumor-only, targeted next-generation sequencing. JCO Precis Oncol 1084–1097 (2020) 1084–97. doi: 10.1200/PO.20.00185 PMC10445788 35050773

[15] Kautto EA Bonneville R Miya J Yu L Krook MA Reeser JW. Performance evaluation for rapid detection of pan-cancer microsatellite instability with MANTIS. Oncotarget (2017) 8:7452–63. doi: 10.18632/oncotarget.13918 PMC5352334 27980218

[16] Cortes-Ciriano I Lee S Park W-Y Kim T-M Park PJ. A molecular portrait of microsatellite instability across multiple cancers. Nat Commun (2017) 8:15180. 28585546 10.1038/ncomms15180 PMC5467167

[17] Wang Y Shi C Eisenberg R Vnencak-Jones CL. Differences in microsatellite instability profiles between endometrioid and colorectal cancers: A potential cause for false-negative results? J Mol Diagn (2017) 19:57–64. doi: 10.1016/j.jmoldx.2016.07.008 27810331 PMC5225298

[18] Wu X Snir O Rottmann D Wong S Buza N Hui P. Minimal microsatellite shift in microsatellite instability high endometrial cancer: a significant pitfall in diagnostic interpretation. Mod Pathol (2019) 32:650–8. doi: 10.1038/s41379-018-0179-3 30443012

[19] Lander ES Linton LM Birren B Nusbaum C Zody MC Baldwin J. Initial sequencing and analysis of the human genome. Nature (2001) 409:860–921. doi: 10.1038/35057062 11237011

[20] Hause RJ Pritchard CC Shendure J Salipante SJ. Classification and characterization of microsatellite instability across 18 cancer types. Nat Med (2016) 22:1342–50. doi: 10.1038/nm.4191 27694933

[21] Rogozin IB Pavlov YI. Theoretical analysis of mutation hotspots and their DNA sequence context specificity. Mutat Res (2003) 544:65–85. doi: 10.1016/s1383-5742(03)00032-2 12888108

[22] Salipante SJ Scroggins SM Hampel HL Turner EH Pritchard CC. Microsatellite instability detection by next generation sequencing. Clin Chem (2014) 60:1192–9. doi: 10.1373/clinchem.2014.223677 24987110

[23] Cerretelli G Ager A Arends MJ Frayling IM. Molecular pathology of lynch syndrome. J Pathol (2020) 250:518–31. doi: 10.1002/path.5422 32141610

[24] Eckert A Kloor M Giersch A Ahmadi R Herold-Mende C Hampl JA. Microsatellite instability in pediatric and adult high-grade gliomas. Brain Pathol (2007) 17:146–50. doi: 10.1111/j.1750-3639.2007.00049.x PMC8095570 17388945

[25] Limpaiboon T. Prognostic significance of microsatellite alterations at 1p36 in cholangiocarcinoma. WJG (2006) 12:4377. doi: 10.3748/wjg.v12.i27.4377 16865781 PMC4087750

[26] Goeppert B Roessler S Renner M Singer S Mehrabi A Vogel MN. Mismatch repair deficiency is a rare but putative therapeutically relevant finding in non-liver fluke associated cholangiocarcinoma. Br J Cancer (2019) 120:109–14. doi: 10.1038/s41416-018-0199-2 PMC6325153 30377340

[27] Goodfellow PJ Billingsley CC Lankes HA Ali S Cohn DE Broaddus RJ. Combined microsatellite instability, MLH1 methylation analysis, and immunohistochemistry for lynch syndrome screening in endometrial cancers from GOG210: An NRG oncology and gynecologic oncology group study. J Clin Oncol (2015) 33:4301–8. doi: 10.1200/JCO.2015.63.9518 PMC4678181 26552419

[28] Wang A McCracken J Li Y Xu L. The practice of universal screening for lynch syndrome in newly diagnosed endometrial carcinoma. Health Sci Rep (2018) 1:e43. doi: 10.1002/hsr2.43 30623082 PMC6266449

[29] Verma L Kane MF Brassett C Schmeits J Evans DG Kolodner RD. Mononucleotide microsatellite instability and germline MSH6 mutation analysis in early onset colorectal cancer. J Med Genet (1999) 36:678–82. PMC1734424 10507723

[30] Arlow T Kim J Haye-Bertolozzi JE Martínez CB Fay C Zorensky E. MutSα mismatch repair protein stability is governed by subunit interaction, acetylation, and ubiquitination. G3 Genes|Genomes|Genetics (2021) 11:jkaa065. doi: 10.1093/g3journal/jkaa065 33793773 PMC8063085

[31] Bartley AN Mills AM Konnick E Overman M Ventura CB Souter L. Mismatch repair and microsatellite instability testing for immune checkpoint inhibitor therapy: Guideline from the college of American pathologists in collaboration with the association for molecular pathology and fight colorectal cancer. Arch Pathol Lab Med (2022) 146:1194–210. doi: 10.5858/arpa.2021-0632-CP 35920830

[32] Shia J Black D Hummer AJ Boyd J Soslow RA. Routinely assessed morphological features correlate with microsatellite instability status in endometrial cancer. Hum Pathol (2008) 39:116–25. doi: 10.1016/j.humpath.2007.05.022 17949789

[33] Shia J. The diversity of tumours with microsatellite instability: molecular mechanisms and impact upon microsatellite instability testing and mismatch repair protein immunohistochemistry. Histopathology (2021) 78:485–97. doi: 10.1111/his.14271 33010064

[34] Bartley AN Hamilton SR Alsabeh R Ambinder EP Berman M Collins E. Template for reporting results of biomarker testing of specimens from patients with carcinoma of the colon and rectum. Arch Pathol Lab Med (2014) 138:166–70. doi: 10.5858/arpa.2013-0231-CPc 23808403

