# Supplementary material for: Improved NGS-based detection of microsatellite instability using tumor-only data
Source: Front Oncol. 2022 Nov 17;12:969238. doi: 10.3389/fonc.2022.969238 (PMC9714634; doi:10.3389/fonc.2022.969238)
Supplement: Supplementary file 1 [file DataSheet_1.pdf]

We aimed to assess the impact of the homopolymer set composition considered in the definition of the MSIscore that maximises MSIdetect's analytical performance.

We first, computed the median homopolymer score at all position using half of the MSS samples, hereafter referred to as the training set. We ranked the mean homopolymer score for each position in the training set and selected the 20% percentile of homopolymers (24 loci) with the highest and lowest homopolymer scores. We refer to these two sets of homopolymers as the highest mean and lowest mean homopolymer set, respectively.

We computed the MSIscore for all MSI-H and the remaining MSS samples using the information from the lowest mean or highest mean homopolymer set. We found that the MSIscore calculated using these different sets of homopolymers are well correlated as illustrated by Supplementary Note Figure 1A. Specifically the correlation between the score obtained using the full restricted set versus the highest mean set was Pearson R=0.993; the correlation between the score obtained using the full restricted set versus the lowest mean set was Pearson R= 0.990; and the correlation between the score obtained using the highest mean set versus the lowest mean set was Pearson R=0.984.

Whereas the distribution of MSIscore for MSI-H and MSS samples is distinct, independently of the set of homopolymers chosen (Supplementary Note Figure 1B), the threshold that maximizes the difference between the two classes varies for different homopolymer set. This is illustrated well by the increase in false positives seen when the threshold defined using the full restricted homopolymer set (defined as described in the manuscript) is used to classify samples based on their MSIscore as computed using the set of homopolymers with the highest mean homopolymer (Supplementary Note Figure 1B).

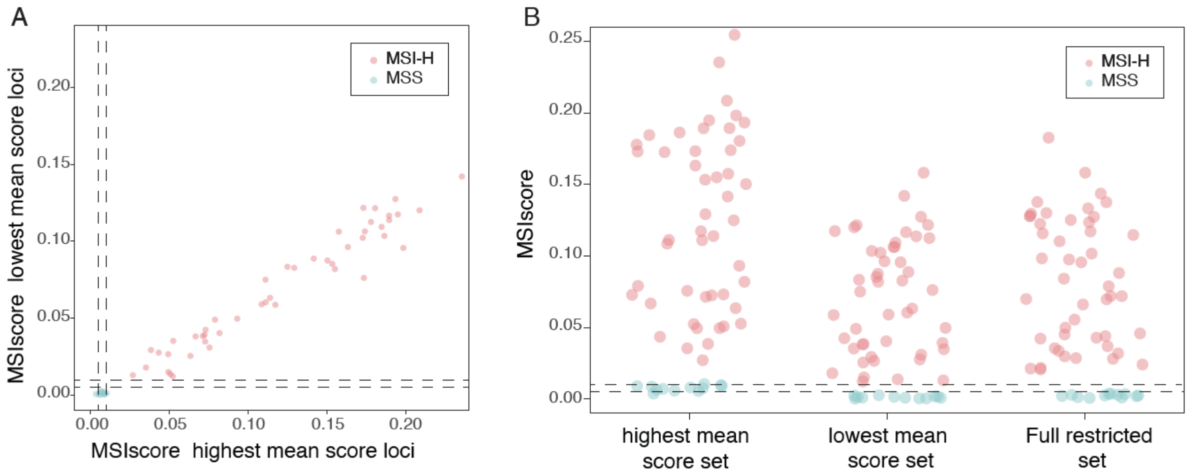

Supplementary Note Figure 1- Homopolymer selection impacts MSIscore classification threshold (A) Relationship between MSIscore obtained for MSI-H (red) and MSS (low) colorectal and endometrial cancer samples using either the 20% percentile of homopolymers with the highest (x-axis) or the lowest (y-axis) mean homopolymer score. Pearson R=0.984 (B) MSIscore (y-axis) computed considering only 20% percentile of homopolymers with the highest (left) or lowest (center) mean homopolymer score; or the full restricted homopolymer set. Samples were grouped based on their respective MSI-PCR and IHC result. Each point corresponds to one sample. Dashed lines indicate the position of MSI-LC and MSI-H classification threshold.
