# Supplementary material for: Improved NGS-based detection of microsatellite instability using tumor-only data
Source: Front Oncol. 2022 Nov 17;12:969238. doi: 10.3389/fonc.2022.969238 (PMC9714634; doi:10.3389/fonc.2022.969238)
Supplement: Supplementary file 2 [file DataSheet_2.pdf]

To test the extend the choice of samples used to build the reference distribution impacts results, we divided, at random, the 24 MSS samples into 3 equally sized groups (8 samples each), hereafter referred to as g1, g2 and g3. We computed reference distributions using data from g1 and g2 and estimated the *MSIscore* of samples in g3 using the 2 different references. We found that the *MSIscore* obtained using reference distribution built using MSS samples in g1 or g2 correlates well (Supplementary Note 2 Figure 1, Pearson R=0.992), indicating the choice of MSS samples included in the reference distribution impacts minimally *MSIscore* results. We restricted this analysis to MSS samples whose score is more strongly impacted by changes in the reference distribution.

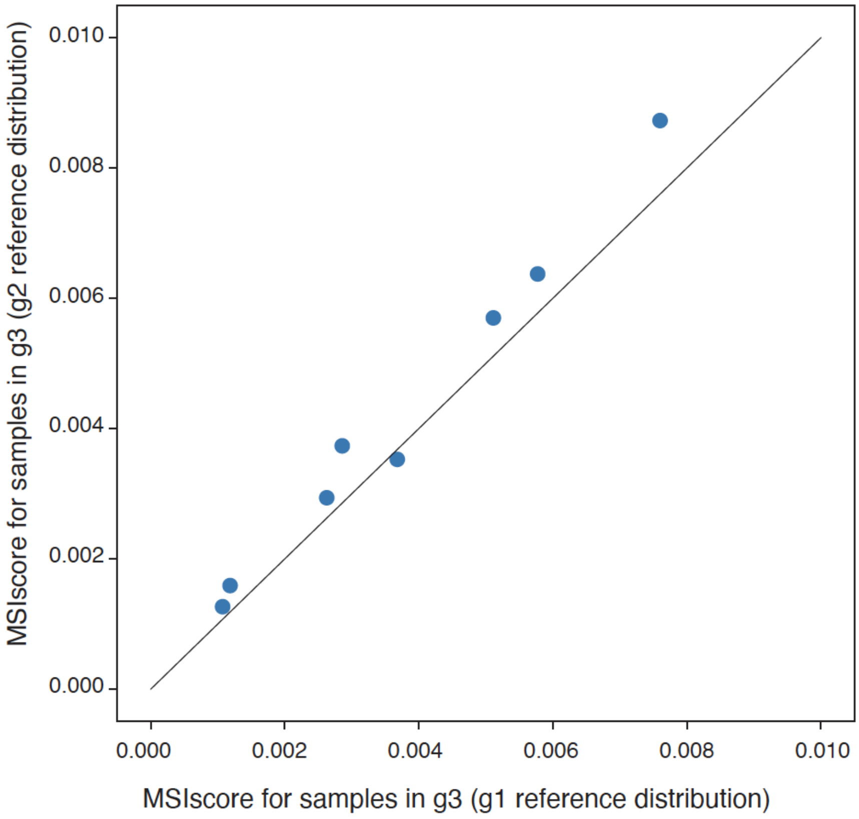

Supplementary Note 2 Figure 1 – Reference sample selection impacts minimally MSIscore*(A) Relationship between MSIscore obtained for MSS in samples group 3 (g3) using reference distribution built using samples in group 1 (g1, X-axis) and group 2 (g2). Each point corresponds to one sample.*
