# Supplementary material for: Improved NGS-based detection of microsatellite instability using tumor-only data
Source: Front Oncol. 2022 Nov 17;12:969238. doi: 10.3389/fonc.2022.969238 (PMC9714634; doi:10.3389/fonc.2022.969238)
Supplement: Supplementary file 3 [file DataSheet_3.pdf]

To establish the minimal number of effective reads, defined hereafter as reads that span the entire homopolymer region plus at least one nucleotide up and downstream of the locus, and estimate the minimal sequencing coverage required per locus for *MSIdetect* we considered a subset of 10 clinical samples with consistent IHC and MSI_PCR results (Table 1) with at least x1500 mean coverage. Homopolymer enriched sequencing libraries for these samples was obtained as described (Materials and Methods). All samples were sequenced on an Illumina NextSeq.

Table 1-Summary of the properties of samples used in the analysis.

Sample	Cancer	IHC	MSI_PCR	Mean Coverage
A34	endometrial_cancer	dMMR	MSI-H	1514
A38	colorectal_cancer	dMMR	MSI-H	3470
A41	colorectal_cancer	dMMR	MSI-H	1969
A50	endometrial_cancer	dMMR	MSI-H	4141
A39	colorectal_cancer	dMMR	MSI-H	3534
A63	endometrial_cancer	pMMR	MSS	4042
A74	endometrial_cancer	pMMR	MSS	4648
A77	colorectal_cancer	pMMR	MSS	4821
A80	colorectal_cancer	pMMR	MSS	7357
A92	colorectal_cancer	pMMR	MSS	4686

For each sample in Table 1 we down-sampled, 10 times, the number of effective reads mapping to each homopolymer to a pre-determined number between 20 and 2000 reads t, and computed, as detailed in the methods, the *MSIscore* for all down-sampled libraries for all samples (Supplementary Note 3 Figure 1).

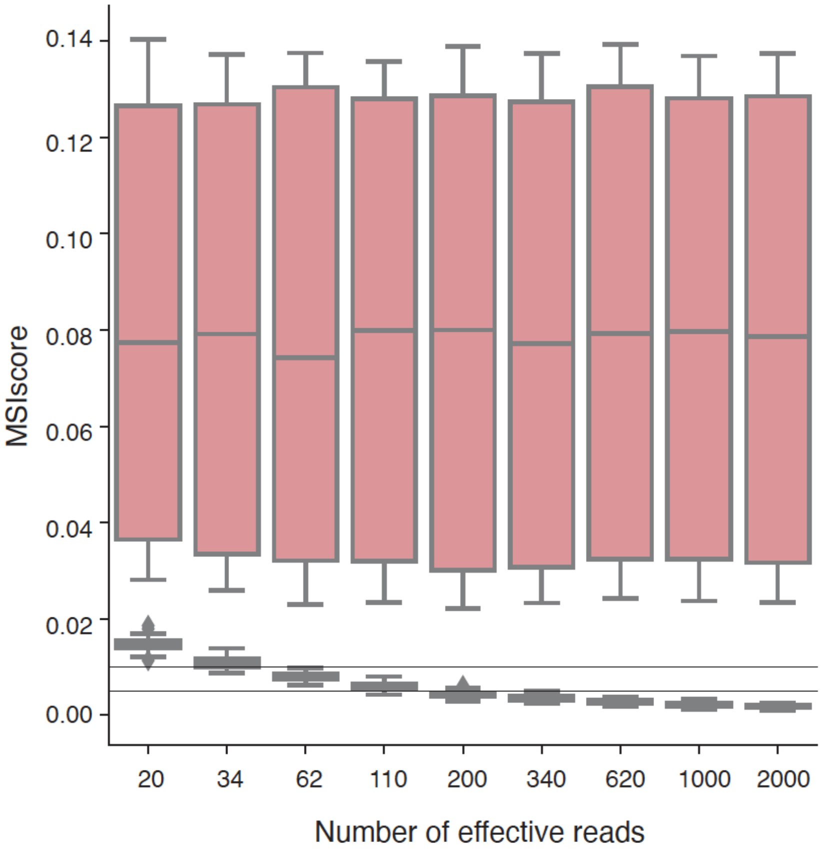

Supplementary Note 3 Figure 1-Distribution of MSIscore according to the effective reads (x-axis) used to compute the homopolymer score for down sampled libraries (10 times) for 5 MSI-H (red) and 5 MSS (grey) samples. Horizontal lines indicate the position of MSIdetect MSI-LC and MSI-H thresholds**.**

As expected, and for all samples lower coverage is associated with a higher *MSIscore* due to limiting sequencing data available for reconstruction of homopolymer length distribution which leads to increased noise.

We conclude that at least 640 effective reads are needed to discriminate accurately between MSS and MSI samples using the current thresholds. Decrease in effective coverage will lead to an increase in the number of false positives. To achieve this number of effective reads, based on the analysis of the NGS data for clinical samples in the present study, requires approximately 1000x coverage.
