# Supplementary material for: Improved NGS-based detection of microsatellite instability using tumor-only data
Source: Front Oncol. 2022 Nov 17;12:969238. doi: 10.3389/fonc.2022.969238 (PMC9714634; doi:10.3389/fonc.2022.969238)
Supplement: Supplementary file 4 [file Table_1.pdf]

**Supplementary Table S1- Number of homopolymers with various % of MSI-H samples with a score higher than the maximal MSS score.**

| % of MSI-H samples with score higher than maximal MSS score | # of homopolymers | | | # of homopolymers common to 3 cancer types |
|---|---|---|---|---|
| | Endometrial | Colorectal | Stomach | |
| 95% | 7 | 551 | 209 | 2 |
| 90% | 37 | 1002 | 448 | 20 |
| 80% | 241 | 1633 | 861 | 136 |
| 65% | 811 | 2218 | 1263 | 485 |
| 50% | 1461 | 2536 | 1537 | 865 |
| 25% | 2339 | 2856 | 1796 | 1455 |
