# Supplementary material for: Improved NGS-based detection of microsatellite instability using tumor-only data
Source: Front Oncol. 2022 Nov 17;12:969238. doi: 10.3389/fonc.2022.969238 (PMC9714634; doi:10.3389/fonc.2022.969238)
Supplement: Supplementary file 5 [file Table_2.pdf]

Supplementary Table S2- Characteristics of different homopolymer sets. Sensitivity at 95% specificity, standard deviation of MSI-H and MSS samples when considering homopolymer sets with a local MSI score in MSI-H samples higher than a given percentile of tumor samples for a given cancer type. Number of homopolymers in the set is indicated inside parenthesis.

percentile (size)	Endometrial Cancer			Colorectal Cancer			Stomach Cancer		
	Sensitivity 95% specificity	Standard Deviation MSS	Standard Deviation MSI-H	Sensitivity 95% specificity	Standard Deviation MSS	Standard Deviation MSI-H	Sensitivity 95% specificity	Standard Deviation MSS	Standard Deviation MSI-H
**95** (2)	95.6%	0.005	0.062	100.0%	0.005	0.062	95.1%	0.005	0.069
**90** (20)	95.6%	0.002	0.038	97.6%#i	0.003	0.047	100.0%	0.003	0.049
**80** (136)	97.1%	0.002	0.034	97.6%	0.002	0.047	100.0%	0.002	0.051
**65** (485)	97.1%	0.002	0.029	97.6%	0.002	0.042	100.0%	0.002	0.045
**50** (865)	95.6%	0.002	0.027	97.6%	0.002	0.040	100.0%	0.002	0.042
**25** (1455)	95.6%	0.002	0.023	97.6%	0.002	0.036	100.0%	0.001	0.038
