# Supplementary material for: Improved NGS-based detection of microsatellite instability using tumor-only data
Source: Front Oncol. 2022 Nov 17;12:969238. doi: 10.3389/fonc.2022.969238 (PMC9714634; doi:10.3389/fonc.2022.969238)
Supplement: Supplementary file 6 [file Table_3.pdf]

Supplementary Table S3- Area Under the Curve (AUC) values for endometrial, colorectal and Stomach (cancer for MSI classification by MSIdetect, mSINGS and MANTIS and mSINGS using restricted homopolymers given the MSI status reported by TGCA in test set

	Endometrial	Colorectal	Stomach
MSIdetect	0.9995	1.000	1.000
mSINGS	0.9998	1.000	1.000
MANTIS	0.9909	1.000	1.000
