# Supplementary figures and images for: Improved NGS-based detection of microsatellite instability using tumor-only data
Source: Front Oncol. 2022 Nov 17;12:969238. doi: 10.3389/fonc.2022.969238 (PMC9714634; doi:10.3389/fonc.2022.969238)

Supplementary Figure 1

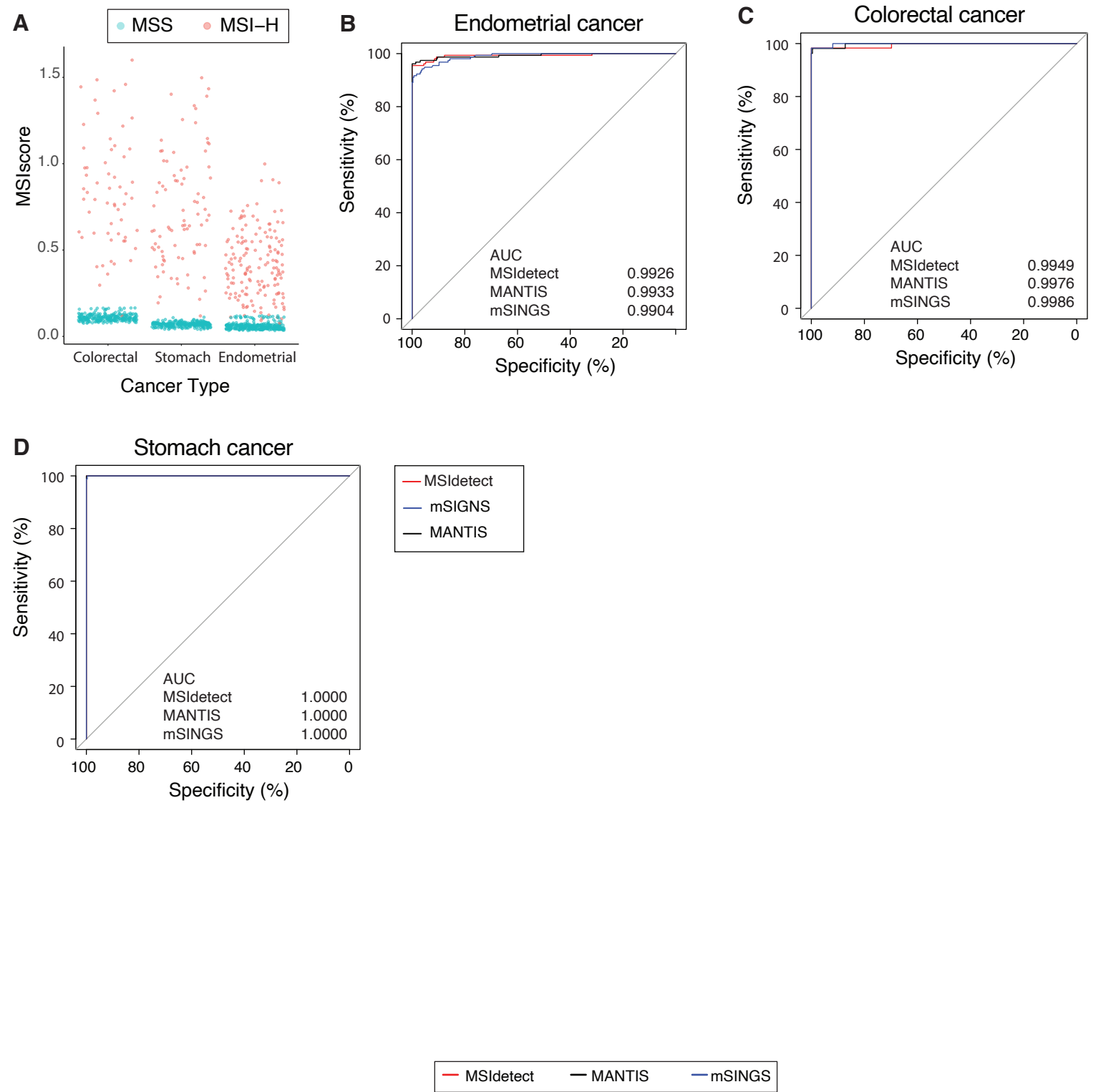

Supplement: Supplementary Figure 1 — (A) Score obtained with MSIdetect, using WES homopolymers in colorectal, stomach and endometrial cancer. Each point corresponds to one sample colored by reported MSI status (refer to legend in the figure). Receiver Operating curves and corresponding Area Under the Curve (AUC) values (in the inset) for endometrial (B), colorectal (C) and stomach (D) cancers for MSI classification by MSIdetect, mSIGNS and MANTIS using WES homopolymers given the MSI status reported by TCGA. [file Image_1.pdf]

Supplementary Figure 2

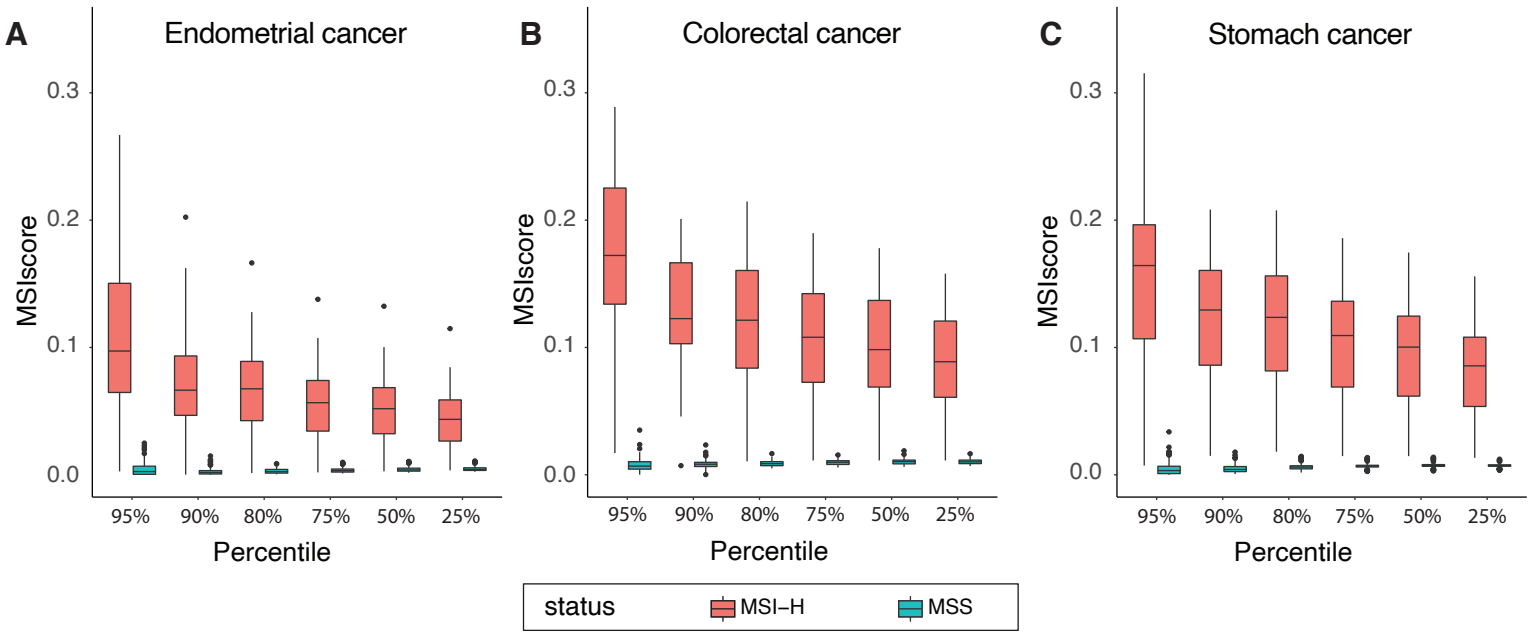

Supplement: Supplementary Figure 2 — Distribution of the MSIscore for microsatellite instability high (MSI-H, red) and stable (MSS, blue) samples in the training set using the different homopolymers combinations in endometrial (A), colorectal (B) and Stomach (C) cancer. [file Image_2.pdf]
